# Anti-NMDA Receptor Encephalitis Misdiagnosed As Generalized Anxiety Disorder: A Case Report

**DOI:** 10.7759/cureus.20529

**Published:** 2021-12-20

**Authors:** Liang Xu, Zheli Chen

**Affiliations:** 1 Department of Geriatric Psychiatry, Huzhou Third People's Hospital, the Affiliated Hospital of Huzhou University, Huzhou, Zhejiang Province, China, Huzhou, CHN

**Keywords:** anti-nmdar encephalitis, hashimoto’s encephalopathy, rapidly progressive dementia, generalized anxiety disorder, autoimmune encephalitis

## Abstract

The autoimmune encephalitis is a rare group of neurological disorders mediated by immune mechanisms. Neuropsychiatric symptoms often occur in the early stages of the disease, so many patients seek treatment in psychiatry for the first time. If psychiatrists lack understanding and vigilance of the disease, it is very easy to be misdiagnosed as various primary psychiatric diseases, thus delaying diagnosis and treatment. Here, we report an older woman with autoimmune encephalitis. In the early stage, the patient was misdiagnosed as having generalized anxiety disorder because of obvious psychiatric symptoms, and then caused the doctor’s vigilance due to rapidly declining cognitive function, autonomic nervous dysfunction, involuntary movement, and other warning symptoms. Autoimmune encephalitis was diagnosed by cerebrospinal fluid examination, and the patient was cured and discharged after a period of immunotherapy.

## Introduction

Autoimmune encephalitis (AE) is a rare type of encephalitis in which the immune system attacks the brain. Anti-N-methyl-D-aspartate receptor (NMDAR) encephalitis is the most common AE [1]. The disease is often acute and more common in children and young people. The main manifestations include abnormal behavior, cognitive impairment, involuntary movement, autonomic dysfunction, and ataxia. The early stage of the disease can be mainly manifested as emotional abnormalities, cognitive impairment, and other mental symptoms, so patients are often first diagnosed in the psychiatric department. Most of the patients with anti-NMDAR encephalitis have a good prognosis if they can be treated in time, so it is crucial to be vigilant and identify the disease as soon as possible [2]. Here, we report a case of anti-NMDAR encephalitis that had been misdiagnosed as a generalized anxiety disorder (GAD) in an elderly patient. In addition, we review the previous relevant literature.

## Case presentation

A 68-year-old female patient was admitted to the hospital on December 2, 2020, due to being “anxious and easily frightened for 3 months, psychomotor retardation, and affected by urinary incontinence for half a month.” The patient had no mental illness before and developed symptoms 3 months before admission. These included waking up early, being nervous and afraid for no apparent reason, and being fearful of leaving the house. The patient was upset, sensitive, and cried occasionally. In addition, the patient needed walking support (e.g., hands on the wall) at home to prevent falling. The patient had been hospitalized at a local mental health center 2 months prior to the present admission, where she was diagnosed with “GAD.” She received paroxetine (20 mg/d), tandospirone (30 mg/d), and oxazepam (15 mg/d). Her symptoms improved, and so she was discharged. She reported that she took the medications regularly according to the instructions. However, half a month prior to admission at our hospital, the patient experienced a relapse characterized by anxiety, fear, small steps while walking, reluctance to come out of her home, speaking less, and being slow to respond, as well as urinary incontinence. In addition, her social skills declined significantly, and the patient could not take care of herself independently. The patient had been diagnosed with type-II diabetes 4 years previously. No other comorbidities were reported. The patient had no abnormalities in her personal history, menstrual history, marriage and childbirth history, or family history.

Physical examination after admission revealed she had normal limb muscle strength and tone. The finger-to-nose, rapid alternating movement, heel-to-shin tests, and Romberg’s sign were normal. However, her gait was not stable and she took small steps. She was negative for pathological signs and meningeal irritation. In a psychological assessment, the patient exhibited clear consciousness, disorientation to place and time, passivity during interactions with few answers to questions, no hallucinations or delusions, a decline in memory and cognition; and a reduction in daily physical activities and energy levels. She felt distraught with a slight tremor in her hands.

**Figure 1:**
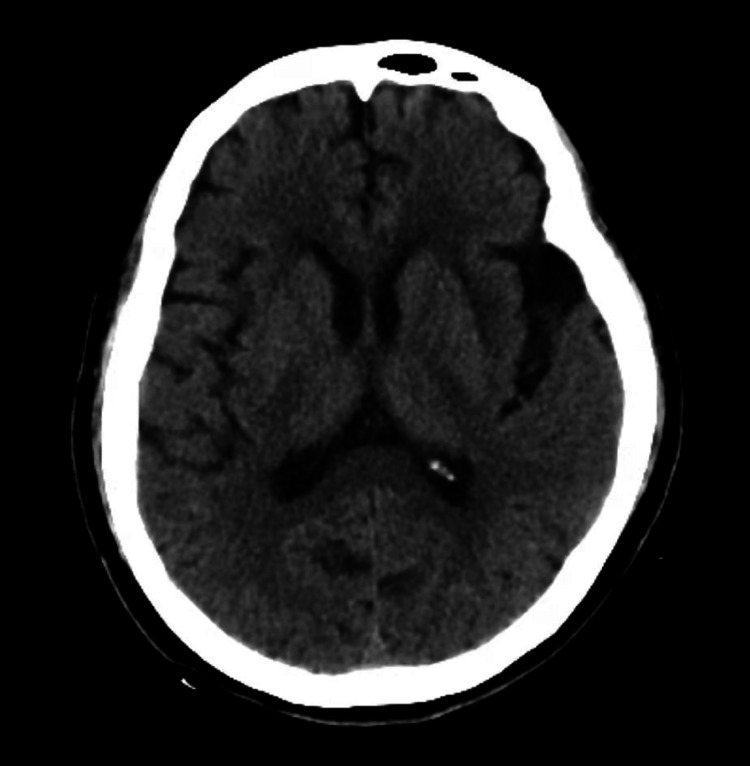
Axial CT scan of the head without contrast showing brain atrophy and leukoaraiosis. CT: computed tomography

**Figure 2:**
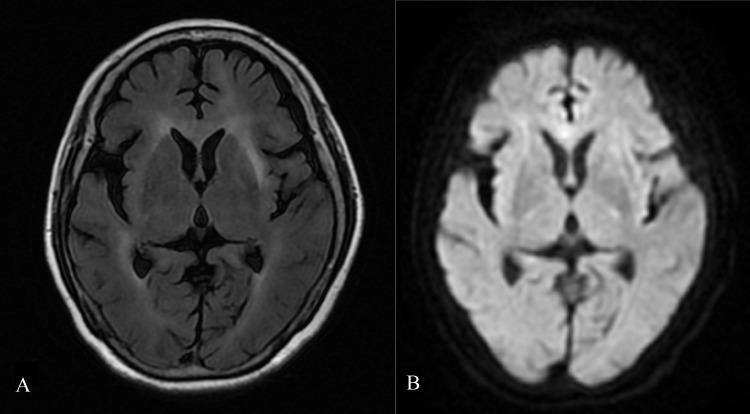
Axial, non-enhanced T2-weighted (A) and DWI (B) MRI image showing brain atrophy and leukoaraiosis. DWI: diffusion-weighted imaging; MRI: magnetic resonance imaging

Her level of thyroid-stimulating hormone (TSH) was 6.280 µIU/mL (0.270-4.200); and she was normal for total T3, total T4, free T3, and free T4. Her level of anti-thyroglobulin antibody (anti-Tg) was 235.60 IU/mL (<115.00), and that of anti-thyroid peroxidase antibody (anti-TPO) was 238.70 IU/mL (<34.00). A color Doppler ultrasound showed diffused thyroid lesions with nodular changes (nodules in the right lobe of the thyroid). Both a CT examination (Figure 1) and an MRI (Figure 2) of the head showed brain atrophy and leukoaraiosis. A chest CT and pelvic ultrasonography were normal. EEG recordings revealed diffuse slow waves. No epileptic activity or extreme delta brush was observed. The patient’s Hamilton Anxiety Scale (HAMA) score was 22, indicating moderate anxiety. Her Hamilton Depression Scale (HAMD) score was 13, indicating a likelihood of depression. Her Mini-Mental State Examination (MMSE) score was 14, indicating dementia.

The differential diagnosis was as follows: 1) rapidly progressive dementia, 2) type-II diabetes, and 3) subclinical hypothyroidism. After admission, the patient was treated with venlafaxine (75 mg/d) and oxazepam (15 mg/d). She showed a rapidly progressive decline in cognitive function, emotional dysregulation, paroxysmal body shaking, slow performance, abnormal gait, constipation, and urinary incontinence. However, the reason for the rapidly progressive dementia was unknown. Thus, lumbar puncture and cerebrospinal fluid examination were performed with the following results: cerebrospinal fluid glucose: 6.30 mmol/L (2.50-4.50); protein: 0.87 g/L (0.15-0.45); and anti-glutamate receptor (NMDA) ratio: 1:3.2 (IgG).

Based on these results, the diagnosis was changed to AE. Accordingly, the patient was transferred to the neurology department and treated with immunoglobulin (IVIG, 20 g/d via intravenous infusion for 5 days) and methylprednisolone (1000 mg/d via intravenous infusion for 3 days). The dosage of methylprednisolone was dropped by half every 3 days (till 48 mg/d) and was given orally. After treatment, the patient showed a gradual recovery in cognitive and emotional function, her body shaking disappeared, and her gait was stable. Her EEG was normal. Both medications were stopped gradually. The patient was discharged 24 days after administration and had resumed a normal life without relapse at a 6-month follow-up.

## Discussion

AE is a relatively rare group of diseases that involve the production of autoantibodies against synapses and neuronal cell surface antigens. Various neuropsychiatric symptoms have been documented for AE [3]. The first study of NMDAR encephalitis patients was published more than 10 years ago [4]. Since then, more than 30 related AEs, characterized by the generation of autoantibodies against synapses and neuronal cell surface antigens, had been discovered. These include anti-NMDAR encephalitis, anti-leucine-rich glioma-inactivated 1 (LGI1) antibody encephalitis, anti-gamma-aminobutyric acid (B) (GABABR) antibody encephalitis, anti-α-amino-3-hydroxy-5-methyl-4-isoxazolepropionic acid receptor (AMPAR) antibody encephalitis, and anti-dipeptidyl peptidase-like protein (DPPX) antibody encephalitis [5-9]. Patients with anti-NMDAR encephalitis show a wide spectrum of neuropsychiatric symptoms including psychotic symptoms, mood disorders, language disorders, memory disorders, seizures, and disturbances of consciousness. These symptoms are typically associated with abnormal movements, autonomic disorders, and unstable respiratory function [10]. Of note, ovarian teratomas are also common in young female patients. For diagnosis, the presence of the anti-GluN1 subunit of the NMDAR antibody in the cerebrospinal fluid and serum has been shown to be useful [11].

In this case study, the main early symptoms were psychiatric, and included insomnia, altered mood, behavioral abnormalities, and gait instability. After being diagnosed with “GAD” and treated with anti-anxiety drugs, the patient’s condition improved for some time, but then she relapsed despite continued treatment with medications. In addition to disordered mood, the patient showed evidence of cognitive decline, autonomic dysfunction, and involuntary movement. Because this occurred in an elderly female patient, it was important to consider the possibility of organic disease. Given the personal history of the patient, several organic diseases were possible, including an infection in the central nervous system, stroke, tumor, AE, and Hashimoto’s encephalopathy (HE). Central nervous system infection and stroke were ruled out based on the following factors: 1) no history or symptoms of an infection; 2) no evidence from blood and cerebrospinal fluid examinations; and 3) no abnormalities via head MRI. The levels of biomarkers for tumor were normal, as were the imaging results at multiple sites, suggesting that the patient did not have a tumor. Thus, AE was the most likely cause. The patient was diagnosed with anti-NMDAR encephalitis based on the diagnostic criteria of Graus et al. (2016) [12] as well as the presence of the following symptoms: disordered mood, cognitive decline, autonomic dysfunction, involuntary movements, and a positive result for the anti-glutamate receptor (NMDA type) in the cerebrospinal fluid. In addition, the patient showed an increased level of TSH, was positive for anti-Tg and anti-TPO, and had diffused thyroid lesions with nodular changes (nodules on the right lobe) as revealed by thyroid color Doppler ultrasound. Anti-NMDAR encephalitis and HE have similar clinical features. An important difference is that there are no characteristic neuron antibodies in serum and cerebrospinal fluid of patients with HE, so HE can be excluded [12,13].

Anti-NMDAR encephalitis is mainly treated by immunotherapy, which includes glucocorticoids, IVIG, and plasma exchange. Patients with tumors can be treated with tumor resection or other anti-tumor therapies [14]. A timely treatment leads to a good prognosis. Since no tumor was found in this case, the patient received glucocorticoids and immunoglobulin, and subsequently showed improvement. No relapse had occurred at the 6-month follow-up.

The early stage of anti-NMDAR encephalitis is characterized by mental and behavioral symptoms, with psychiatric symptoms occurring first in more than 40% of all patients. Due to psychological symptoms such as hallucination, agitation, and affective disorders, around one-third of patients are first assessed at a psychiatric department. Thus, anti-NMDAR encephalitis can be easily misdiagnosed as a functional psychological disorder, resulting in a delay in appropriate treatment. The patient in this case study was misdiagnosed as having GAD due to early stage symptoms [15,16]. However, four lines of evidence indicated the possibility of a neurological disorder. First, the development of multiple psychological symptoms such as insomnia, disordered mood, and decline in cognition in a short period of time in this elderly patient with no history of mental disorders was not in line with the characteristics of psychiatric disorders. Second, her rapid decline in cognition and time and location disorientation indicated that progressive dementia should be considered for this case. Third, in addition to psychiatric symptoms, the patient also showed symptoms of neurological disorders such as autonomic dysfunction, gait instability. These symptoms can serve as warning signs and should attract attention from doctors [17]. Last, although the patient’s condition improved following the early treatment for a mood disorder, she quickly relapsed. Accordingly, she was further examined using EEG, MRI, and cerebrospinal fluid analysis to enable a correct diagnosis.

## Conclusions

In conclusion, the early stage of anti-NMDAR encephalitis is associated with various psychiatric symptoms. In addition to these rapidly developing mental symptoms in the early stage, other warning signs for anti-NMDAR encephalitis include a rapid decline in cognition, autonomic dysfunction, gait instability, involuntary movements, poor response to psychotropic drugs, and relapses following drug treatment. Timely treatment often leads to a good prognosis in patients.
